# Low-Density Lipoprotein Receptor Contributes to β-Carotene Uptake in the Maternal Liver

**DOI:** 10.3390/nu8120765

**Published:** 2016-11-29

**Authors:** Varsha Shete, Brianna K. Costabile, Youn-Kyung Kim, Loredana Quadro

**Affiliations:** 1 Department of Food Science and Rutgers Center for Lipid Research and New Jersey Institute of Food Nutrition and Health, Rutgers University, New Brunswick, NJ 08901, USA; varsha.shete@gmail.com (V.S.); bkc2117@cumc.columbia.edu (B.K.C.); ykkim5@sebs.rutgers.edu (Y.-K.K.); 2 Department of Pathology, Northwestern University, Feinberg School of Medicine, Chicago, IL 60611, USA; 3 Columbia University, Integrated Graduate Program, New York, NY 10032, USA

**Keywords:** β-carotene uptake, vitamin A, LDL receptor, maternal liver

## Abstract

Vitamin A regulates many essential mammalian biological processes, including embryonic development. β-carotene is the main source of vitamin A in the human diet. Once ingested, it is packaged into lipoproteins, predominantly low-density lipoproteins (LDL), and transported to different sites within the body, including the liver and developing tissues, where it can either be stored or metabolized to retinoids (vitamin A and its derivatives). The molecular mechanisms of β-carotene uptake by the liver or developing tissues remain elusive. Here, we investigated the role of the LDL receptor (LDLr) in β-carotene uptake by maternal liver, placenta and embryo. We administered a single dose of β-carotene to *Ldlr^+/−^* and *Ldlr^−/−^* pregnant mice via intraperitoneal injection at mid-gestation and monitored the changes in β-carotene content among maternal lipoproteins and the liver, as well as the accumulation of β-carotene in the placental–fetal unit. We showed an abnormal β-carotene distribution among serum lipoproteins and reduced hepatic β-carotene uptake in *Ldlr^−/−^* dams. These data strongly imply that LDLr significantly contributes to β-carotene uptake in the adult mouse liver. In contrast, LDLr does not seem to mediate acquisition of β-carotene by the placental–fetal unit.

## 1. Introduction

Vitamin A plays a pivotal role in several biological functions including the normal growth and development of the mammalian embryo [1] by regulating the transcription of genes critical for embryogenesis through its active form retinoic acid [2,3]. β-carotene, the most abundant and best-characterized dietary carotenoid [4], serves as a major source of retinoids (vitamin A and its derivatives) in adult and developing tissues, via a predominant symmetric cleavage mediated by β-carotene-15,15′-oxygenase (BCO1) [5,6]. In addition, asymmetric cleavage of β-carotene by β-carotene-9′,10′-oxygenase (BCO2), yields β-apo-10′-carotenal that can be also converted into retinoids by BCO1 [5,7], even though the contribution of this latter cleavage pathway to the tissue retinoid pool seems to be negligible, at least in adult tissues [5].

In the bloodstream of humans and a number of animal models, β-carotene has been found to be associated with all types of lipoproteins to a varying degree, with the low-density lipoprotein (LDL) being the main carrier of this carotenoid, transporting about 60%–70% of total serum β-carotene [8,9,10]. ApoB- and apoE-containing lipoproteins, mostly LDL and very low-density lipoprotein (VLDL) particles, are endocytosed by the LDL receptor (LDLr) to facilitate cellular lipid acquisition [11]. Indeed, cellular uptake of cholesterol is disrupted in the absence of LDLr in adult tissues, and mice lacking this receptor show abnormally high levels of serum LDL-cholesterol and, overall, slower lipoprotein clearance from the bloodstream [12]. However, the role of LDLr in the cellular uptake of β-carotene remains to be explored.

Given that LDLr is prominently expressed in the liver as well as in the placenta [13,14,15,16] and the embryo [8,17], we hypothesized that LDLr could contribute to β-carotene uptake in these tissues. In this study, upon administration of a single dose of β-carotene to pregnant mice lacking one or both copies of the *Ldlr* gene, we showed that LDLr participates in the uptake of β-carotene by the maternal liver but not by embryo or placenta.

## 2. Materials and Methods

### 2.1. Mice and β-Carotene Administration

Mice lacking either one or both copies of the LDL receptor gene (*Ldlr*) were used (*Ldlr*^+/−^ and *Ldlr*^−/−^, respectively). All mice, obtained from The Jackson Laboratory (Bar Harbor, ME, USA. Strain name: B6.129S7-*Ldlr*^tm1Her^/J, Background: C57BL/6J) were maintained and bred on a standard nutritionally complete chow diet containing 18 IU of vitamin A/g diet and 1.2 ppm of carotenoids (Prolab Isopro RMH3000 5p75; W.F. Fisher and Son, Inc., Somerville, NJ, USA). Genotype was confirmed by PCR according to vendor’s protocol. At approximately three months of age, females were mated according to one of the following schemes: (1) *Ldlr*^+/−^ females (*n* = 11) mated with *Ldlr*^−/−^ males; (2) *Ldlr*^−/−^ females (*n* = 11) mated with *Ldlr*^+/−^ males. In both instances, each dam carried both homozygous and heterozygous embryos. Onset of pregnancy (0.5 days post coitum, dpc) was established by the occurrence of a vaginal plug, the morning after the mating. Diet and water were available to the animals on an ad libitum basis. Mice were maintained on a 12:12 light/dark cycle (light on from 7 a.m. to 7 p.m.). All experiments were conducted in accordance with the National Institutes of Health Guide for the Care and Use of Laboratory Animals [18] and were approved by the Rutgers University Institutional Committee on Animal Care (05-009).

β-carotene was administered to the dams by intraperitoneal (IP) injection at mid-gestation, following a protocol previously established in our laboratory to circumvent the high mouse intestinal BCO1 cleavage activity and yield detectable intact β-carotene in the maternal bloodstream without signs of embryonic vitamin A toxicity [8,19]. An emulsion was prepared by adding β-carotene (Type II, Sigma Aldrich) to a mixture of ethanol, cremophor (Sigma, St. Louis, MO, USA) and PBS (1:11:18 ratio), under yellow light with vortexing. Spectrophotometry at 450 nm was used to determine the concentration of the emulsion (~3.5 g/L). Seven dams of each genotype were injected with a dose of ~35 μg of β-carotene/g of body weight in a volume of ~250 μL at 13.5 dpc. Four dams of each genotype injected with vehicle (same volume) served as controls. The dams were constantly fed throughout the experiment. All animals were euthanized at 14.5 dpc by CO_2_ asphyxiation followed by exsanguination between 9 a.m. and 11 a.m. Maternal serum and liver, as well as placenta and embryos were collected and stored at −80 °C until further analyses. Yolk sac and/or a small piece of embryonic tissue were used to determine the embryonic genotype.

### 2.2. Serum Lipoprotein Isolation

Mouse serum was subjected to density gradient ultracentrifugation to separate VLDL + chylomicron fraction (*d* < 1.006 g/mL), LDL + IDL fraction (*d* = 1.023–1.06 g/mL) and HDL fraction (*d* = 1.063–1.21 g/mL), as previously described [8,20]. Briefly, for the first centrifugation step, 100 µL of the serum was overlaid with 100 µL of 0.9% saline solution (*d* = 1.004 g/mL) and centrifuged at a speed of 70,000 *g* in Beckman TLA 120.1 rotor (Beckman Coulter Inc., Brea, CA, USA) for 4 h. The top layer containing VLDL + chylomicrons was removed using a 100 µL Hamilton glass syringe (Hamilton Company, Reno, NV, USA). The remaining 100 µL was mixed with 100 µL of KBr solution (*d* = 1.12 g/mL) and centrifuged at the same speed for 18 h. The top 100 µL of the LDL + IDL fraction and the bottom 100 µL of the HDL fraction were separated.

### 2.3. Cholesterol and Triglyceride Measurements in the Lipoprotein Fractions

All the isolated serum lipoprotein fractions, as well as total sera, were used to determine total cholesterol (Cholestrol E kit, Wako Diagnostics, Mountain View, CA, USA) and triglycerides (Infinity Triglyceride Kit, Thermo Fisher Scientific Inc., Middeltown, VA, USA), according to the manufacturer’s procedures.

### 2.4. HPLC Analysis of Retinol, Retinyl Ester and β-Carotene

Reverse-phase HPLC analysis was performed to measure serum and tissue retinoid and β-carotene concentrations, as previously described [6,21]. Briefly, tissues (100–200 mg) were homogenized in PBS. Half of the tissue homogenate, or 100 µL in the case of serum, were used to extract retinoids [21]. The other half of the tissue homogenate, or 100 µL of serum or lipoprotein fraction, were used to extract β-carotene. Retinol, retinyl ester, and β-carotene were separated on a C18 column (Hichrom Limited, Reading, UK) using acetonitrile, methanol, and methylene chloride (70:15:15, *v/v*) as the mobile phase. A Dionex Ultimate 3000 HPLC system and a computerized data analysis workstation with Chromeleon software were used. Retinol, retinyl esters and β-carotene were identified by comparing retention times and spectral data of experimental compounds with those of authentic standards. Retinyl acetate (Sigma, St. Louis, MO, USA; for retinoids) and echinenone (CaroteNature, Ostermundigen, Switzerland; for β-carotene) were added as internal standards. Detection limits are as follows: for retinoids-serum <0.1 ng/dL and tissues <1 ng/g; for β-carotene-serum <1 ng/dL and tissues 10 ng/g).

### 2.5. RNA Extraction, cDNA Synthesis, and Quantitative Real-Time PCR (qPCR)

Total RNA was extracted from a sample of liver and individual placentas and embryos using TRIzol^®^ (Invitrogen Corp., Carlsbad, CA, USA), according to the manufacturer’s instructions. RNA was reverse transcribed to cDNA using the instructions and reagents (random hexamer primers were used) from Transcriptor First Strand cDNA Synthesis kit (Roche Diagnostics, Indianapolis, IN, USA). A LightCycler 480 SYBR Green I Master kit (Roche Diagnostics, Indianapolis, IN, USA) was used for qPCR. For the qPCR experiments, 300 nM of each specific primer (final concentration), together with 10–50 ng of cDNA equivalent of the total RNA was used. β-actin was used as the reference gene. All samples were run in duplicates or triplicates. NRTC and the non-template were included within each experiment as controls. After enzyme activation (10 min, 95 °C), 35–40 PCR amplification cycles were performed: 10 s at 95 °C, 20 s at 58 °C, and 30 s at 72 °C. At the end of each run, samples were heated to 95 °C with a temperature transition rate of 0.11 °C/s to construct dissociation curves. From the instrument, cycle thresholds (Ct) were obtained for each sample for each gene of interest. To determine changes in gene expression, the ΔΔCt method was used. The expression of each gene relative to the calibrator was calculated using 2(−ΔΔCt). Gene expression changes were expressed as mRNA fold change from the control group (*Ldlr*^+/−^ + vehicle). Primers and amplicon size are as previously reported [8,19].

### 2.6. Western Blot Analysis

Serum RBP levels were assessed by Western blot analysis according to standard procedures. An amount of 1 μL of serum samples (diluted 1:10) from *Ldlr*^+/−^ and *Ldlr*^−/−^ mice was loaded onto a 12% SDS-PAGE. Polyclonal rabbit anti-mouse RBP (Adipogen International, San Diego, CA, USA) and polyclonal rabbit anti-mouse albumin (Abcam, Cambridge, MA, USA) were used for immunodetection. Albumin was used as a loading control. The quantification of the membranes was completed by densitometry analysis with Quantity One Program (Bio-Rad Laboratories, Hercules, CA, USA). Western blot analysis was repeated twice.

### 2.7. Statistical Analyses

The 2X2 experimental design yielded four experimental groups consisting of *Ldlr*^+/−^ and *Ldlr*^−/−^ embryos from both *Ldlr*^+/−^ and *Ldlr*^−/−^ dams. Normally distributed data were analyzed by 2-way ANOVA followed by a Student’s *t*-test or one-way ANOVA. Data with a non-normal distribution were analyzed by a Kruskal–Wallis test, followed by a Mann–Whitney test. Analyses were performed with GraphPad Prism^®^ version 5.0c. A *p*-value < 0.05 was used to establish statistical significance.

## 3. Results

### 3.1. β-Carotene and Retinoid Concentrations in Maternal Serum and Lipoprotein Particles

**Figure 1:**
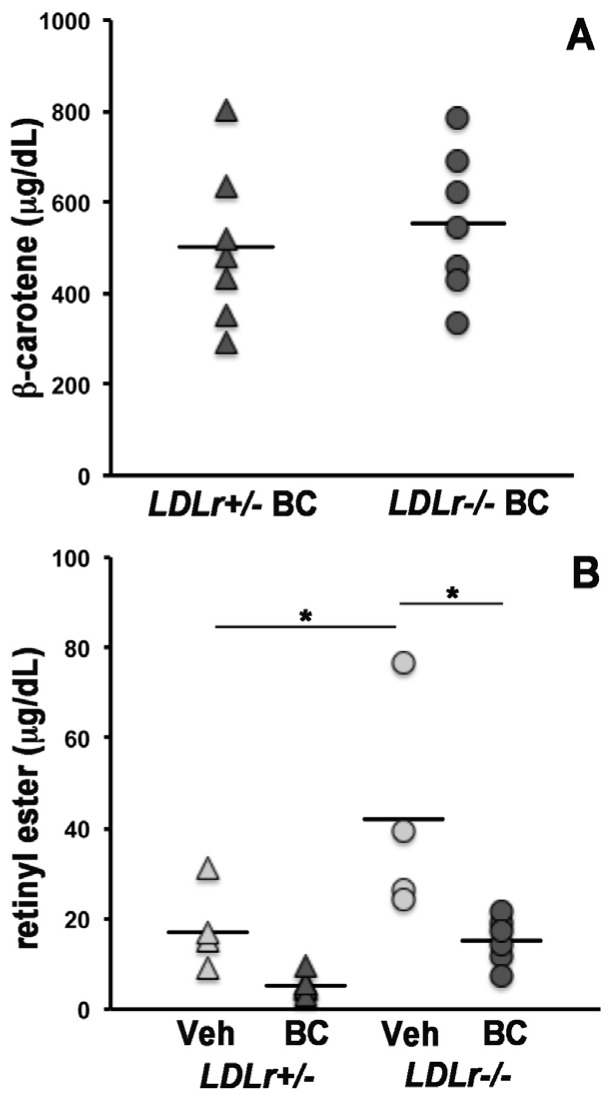

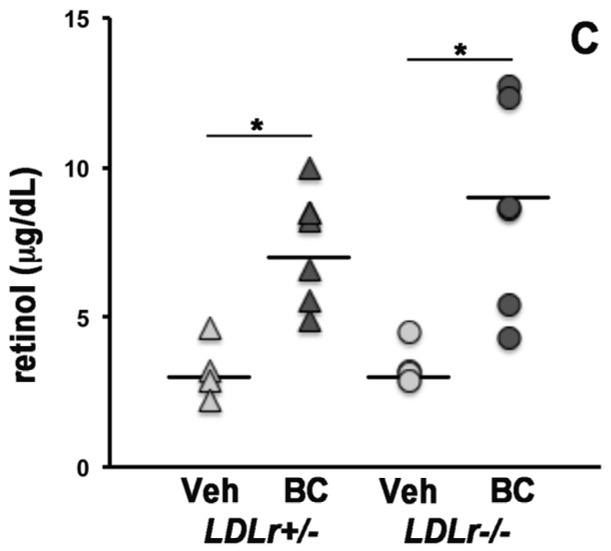
β-carotene and retinoid levels in the serum of *Ldlr^+/−^* and *Ldlr^−/−^* pregnant mice administered β-carotene at 13.5 dpc. Analysis by HPLC and concentrations expressed as μg/dL. (**A**) β-carotene (BC); *n* = 7/group; (**B**) retinyl ester; *n* = 4 for *Ldlr^+/−^* + vehicle (veh), *n* = 7 for *Ldlr^+/−^* + BC, *n* = 4 for *Ldlr^−/−^* + veh, and *n* = 6 for *Ldlr^−/−^* + BC; (**C**) retinol; *n* is same as in (**B**). Individual measurements are shown. Statistical analysis by Kruskal–Wallis test; * *p* < 0.05.

**Figure 2:**
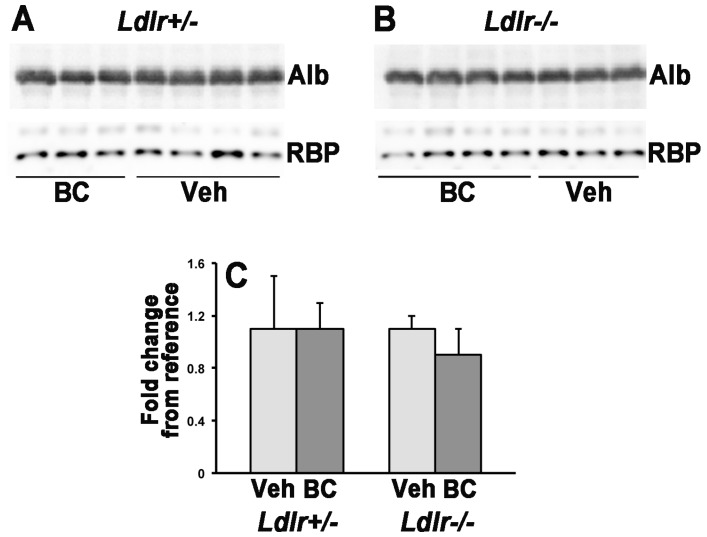
Representative RBP Western blots for *Ldlr^+/−^* (**A**) and *Ldlr^−/−^* (**B**) serum samples. Procedures as indicated in the Materials and Methods section. RBP, 21 kDa; Albumin (Alb), 65 kDa; (**C**) Quantification of the Western blot membranes. Results (expressed as mean ± SD) are shown as a fold change from the reference vehicle (Veh) group within each respective genotype. BC, β-carotene.

In order to establish the contribution of LDLr to the uptake of circulating β-carotene by the maternal and developing tissues, we crossed *Ldlr*^+/−^ females with *Ldlr*^−/−^ males and vice versa. This experimental design, in which each dam carried both *Ldlr*^+/−^ and *Ldlr*^−/−^ embryos, enabled us to evaluate the effects of the lack of placental and embryonic *Ldlr* in the context of a lipoprotein metabolism more or less compromised depending upon the number of *Ldlr* knockout alleles in the dam [12]. We first asked whether the lack of the *Ldlr* gene would impact the concentration of β-carotene and retinoids in maternal serum. As previously reported, circulating β-carotene was undetectable in vehicle-injected control dams [8,17]. In the dams administered the provitamin A carotenoid, β-carotene concentrations did not significantly differ between *Ldlr*^+/−^ and *Ldlr*^−/−^ groups (Figure 1A). Serum retinyl ester levels were higher in *Ldlr*^−/−^ dams compared to *Ldlr*^+/−^ dams in the vehicle injected group (Figure 1B). These data are consistent with earlier reports of slower clearance of these lipophilic moieties from the circulation in the absence of the LDL receptor [12]. Despite a similar trend, retinyl esters levels were not significantly different in the β-carotene injected groups (Figure 1B). Moreover, a trend of reduced serum retinyl ester levels between vehicle- and β-carotene-injected dams also existed but it reached statistical significance only in the *Ldlr*^−/−^ dams (Figure 1B). Interestingly, maternal β-carotene administration resulted in higher concentrations of serum retinol, regardless of the genotype (Figure 1C). However, Western blot analysis indicated that these higher circulating retinol levels were not accompanied by increased concentrations of serum retinol-binding protein (RBP) (Figure 2), the sole specific carrier of retinol in the bloodstream [22].

β-carotene concentrations were next determined in lipoprotein fractions (VLDL + chylomicrons; LDL + IDL; and HDL) isolated from sera of *Ldlr*^+/−^ and *Ldlr*^−/−^ dams. Enrichment of each fraction with specific types of lipoproteins was confirmed by measuring the cholesterol and triglyceride content of each isolated fraction. As previously reported [12], cholesterol levels were elevated in whole serum, as well as in VLDL + chylomicrons and LDL + IDL fractions of *Ldlr*^−/−^ dams compared to the heterozygous females (Table 1).

Furthermore, while maternal total serum TG levels were not different between the two genotypes, *Ldlr*^−/−^ dams showed increased TG concentrations in the VLDL + chylomicrons fraction compared to *Ldlr*^+/−^ females (Table 1). As previously reported in wild-type dams [8], HPLC analysis of β-carotene levels in lipoproteins confirmed that β-carotene was incorporated in all the lipoprotein fractions of both *Ldlr*^+/−^ and *Ldlr*^−/−^ dams to a varying degree (Table 1). When genotypes were compared, VLDL + chylomicron of *Ldlr*^−/−^ dams showed significantly higher levels of β-carotene compared to the same fraction of *Ldlr*^+/−^ dams (Table 1). Overall, these data suggest that β-carotene distribution within serum lipoproteins was altered in *Ldlr*^−/−^ dams, a phenomenon that was classically observed in the case of cholesterol.

### 3.2. Lack of LDLr Affects β-Carotene Accumulation in Maternal Liver

Since the liver is a prominent storage site for β-carotene within the body [23], we asked whether the lack of *Ldlr* would affect the uptake of circulating β-carotene by the maternal liver. β-carotene was undetectable in the liver of the dams administered vehicle (Table 2), as previously reported [8,17,24]. Interestingly, a ~50% reduction in hepatic β-carotene concentration was observed in *Ldlr*^−/−^ dams compared to *Ldlr*^+/−^ dams (Table 2). In contrast, maternal liver retinol and retinyl esters levels were not significantly different between the two genotypes, regardless of the treatment (Table 2). These data strongly suggest that hepatic clearance of β-carotene containing lipoproteins was reduced in the absence of LDLr.

**Figure 3:**
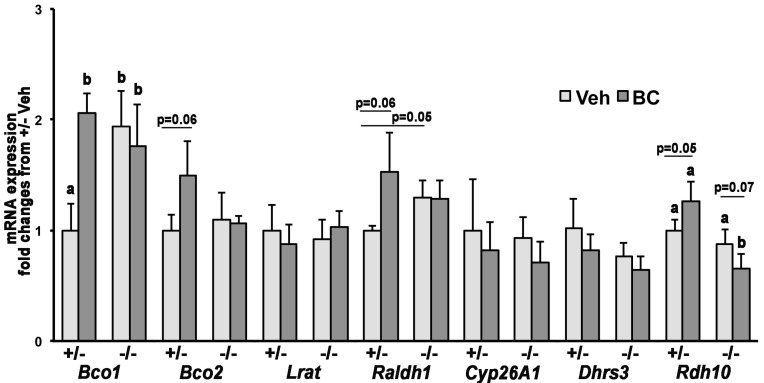
Hepatic mRNA expression of key genes of carotenoid and retinoid metabolism in *Ldlr^+/−^* (+/−) and *Ldlr^−/−^* (−/−) pregnant mice administered β-carotene at 13.5 dpc. Analysis by qPCR. Data are presented as mean ± SD (*error bars*) of duplicate determinations and are representative of three independent determinations; *n* = 3 dams/group. Statistical analysis by ANOVA. Labeled means (within each gene) without a common letter differ, *p* < 0.05; borderline significant differences (*p* < 0.1) between two groups are indicated and the respective p value is given.

To exclude that the lower β-carotene concentration in *Ldlr*^−/−^ livers resulted from enhanced carotenoid breakdown, we measured *Bco1* and *Bco2* mRNA levels. Only in *Ldlr*^+/−^ livers, β-carotene availability significantly increased mRNA levels of *Bco1* (Figure 3). Moreover, in *Ldlr*^−/−^ livers, baseline *Bco1* levels (i.e., in vehicle-injected group) were elevated when compared to *Ldlr*^+/−^ livers. However, β-carotene availability effaced this effect between the genotypes (Figure 3). Hepatic *Bco2* mRNA levels showed only a trend towards an increase in *Ldlr*^+/−^ dams upon β-carotene administration (*p* = 0.06), but there was no treatment- or genotype-dependent difference in its transcription levels (Figure 3). Despite no unequivocal correlation having been established between trascription levels of the β-carotene cleavage enzymes and their activity, it is known that their transcription is regulated by carotenoid availability, as well as by retinoic acid (in the case of *Bco1*) [19]. Thus, changes in their mRNA expression may reflect homeostatic control of the flux of retinoids and carotenoids within tissues, responding to changes in β-carotene and vitamin A status. Overall, our data suggest that when β-carotene is available, such homeostatic regulatory mechanisms are similar in the liver of *Ldlr*^+/−^ and *Ldlr*^−/−^ mice. qPCR analysis of the key regulators of tissue retinoid homeostasis revealed a significant reduction in *Rdh10* mRNA in the *Ldlr*^−/−^ compared to *Ldlr*^+/−^ dams upon β-carotene treatment (Figure 3). Moreover, β-carotene resulted in a trend towards increased mRNA levels of *Rdh10* and *Raldh1* in the liver of *Ldlr*^+/−^ dams (Figure 3), overall suggesting a potential enhancement of the synthesis of retinoic acid from retinol in these heterozygous livers. However, given the lack of significant differences in hepatic retinol and retinyl ester concentrations between the two genotypes (Table 2), it is difficult to establish the biological significance of these changes, at this time. All together, these results support the hypothesis that the reduction in maternal hepatic β-carotene accumulation was due to lack of LDLr and not to enhanced metabolism of the provitamin A carotenoid in *Ldlr*^−/−^ livers.

### 3.3. Lack of LDLr Does Not Impact β-Carotene Uptake by the Developing Tissues

We next investigated the effect of the lack of Ldlr on circulating β-carotene uptake by the placental–fetal unit. In the murine placenta, exchange of nutrients and gas takes place in the syncytiotrophoblast layer, which is of embryonic origin [25]. Therefore, we refer to the syncytiotrophoblast genotype (identical to the embryo’s and yolk sac’s genotype) as the placental genotype. Placentas and embryos of both Ldlr genotypes (Ldlr^+/−^ and Ldlr^−/−^) were obtained from both Ldlr^+/−^ and Ldlr^−/−^ dams treated with vehicle or β-carotene, resulting in eight experimental groups (Table 3). β-carotene was not detectable in placentas or embryos from vehicle-injected dams, consistent with our earlier reports [8,17,24]. Upon maternal supplementation, β-carotene levels did not differ among groups, regardless of the maternal or placental/embryonic genotype, thus excluding a potential critical contribution of LDLr to β-carotene accumulation in the placental–fetal unit (Table 3). Placental or embryonic retinol and retinyl ester levels were also not different among the eight experimental groups (data not shown), suggesting that neither maternal nor placental/embryonic LDLr influences the retinoid status of the placental/fetal unit.

## 4. Discussion

It is well established that dietary β-carotene, circulating in the bloodstream of humans and animals in association with lipoproteins, can serve as an in situ source of retinoids once taken up by both adult and developing tissues [5,6]. However, the mechanisms that regulate cellular uptake of β-carotene are poorly understood. Most of the research in this area has focused on the mechanisms of β-carotene uptake by the enterocytes and the retinal pigment epithelium (RPE) cells. In both instances, the scavenger receptor B1 (SR-B1) has been shown to mediate absorption of β-carotene in vivo and in vitro [26,27,28,29,30,31]. Specifically, in the enterocytes, this function is regulated by a negative feedback mechanism, depending upon the vitamin A status and mediated by an intestinal specific homeobox protein (ISX) transcriptionally regulated by retinoic acid [30,32]. Thus, when intestinal retinoic acid levels are high, as in the case of excessive intake of vitamin A, ISX levels increase to downregulate the mRNA expression of *Srb-1* and reduce intestinal absorption of dietary β-carotene [30,32]. However, the role of SR-B1 and other lipoprotein receptors in this process, especially in the liver, one of the major sites of storage and metabolism of the provitamin A, remains elusive. Similarly, it is not fully understood how the placenta or the developing embryo acquire β-carotene from the maternal circulation. Based on the findings of von Lintig and colleagues [30,32], the lack of transcriptional regulation of placental and embryonic *Scarb1* by dietary β-carotene [8,17,24], strongly suggest that SR-B1 may not play a major role in the uptake of the provitamin A carotenoid by the developing tissues. In contrast, LDL-receptor related protein 1 (LRP1) and very low-density lipoprotein receptor (VLDLr), emerged as potential modulators of placental β-carotene uptake, depending upon the maternal vitamin A dietary intake and status [8,17,24].

Given that the majority of serum β-carotene circulates in association with LDL, in both humans and experimental animal models [8,9,10,33], we investigated the role of LDLr in the regulation of β-carotene uptake by adult (maternal liver) and developing tissues (placenta and embryo). LDLr is the main receptor for apoB- and apoE-containing lipoprotein particles [12,34], and *Ldlr*^−/−^ mice show elevated total serum cholesterol, high VLDL and LDL cholesterol and high number of LDL and VLDL particles in the circulation compared to wild-type mice [12,34]. They also show higher levels of serum retinyl esters carried in the bloodstream by lipoproteins [12]. Our study confirmed that *Ldlr*^−/−^ mice have reduced clearance of serum cholesterol and retinyl esters. However, when β-carotene is available (at relatively high levels), the concentration of serum retinyl esters, likely associated with circulating lipoproteins, is significantly reduced, at least in the *Ldlr*^−/−^ dams. These data suggest a potential competitive mechanism between β-carotene and retinyl esters for the incorporation into lipoprotein particles, at least in this model of impaired lipoprotein clearance. The mechanism of incorporation of retinoids and carotenoids into lipoprotein particles is currently unknown, although recent data from our lab strongly suggest that microsomal triglyceride transfer protein (MTP) may mediate this process in the case of β-carotene [19]. Interestingly, a single β-carotene administration results in increased maternal serum retinol concentrations in both *Ldlr*^+/−^ and *Ldlr*^−/−^ dams without changes in serum levels of RBP. Whether the higher serum retinol is transported within lipoproteins and is a consequence of the reduced serum retinyl ester concentrations upon β-carotene treatment remains to be established. However, this seems to be a genotype-dependent effect, as it was not previously observed in wild-type pregnant dams administered β-carotene under the same experimental protocol [8,17].

In contrast to wild-type dams in which the LDL + IDL fraction contained the highest amount of β-carotene [8], under the same experimental condition, the provitamin A carotenoid was similarly distributed in LDL + IDL and HDL fractions in both *Ldlr*^+/−^ and *Ldlr*^−/−^ dams. It is of note that even the absence of a single copy of the *Ldlr* gene reduces lipoprotein clearance [35,36]. Thus, despite the rather limited sample size of our lipoprotein analysis, our data suggest that the absence of one or both copies of *Ldlr* alters β-carotene and retinoid incorporation within serum lipoproteins.

Whether the above-mentioned altered proportion of circulating vitamin A forms in mice lacking LDLr impacts retinoid content of the liver—the main body storage site of vitamin A—is still unclear. Hepatic retinoid levels were undistinguishable between vehicle- and β-carotene-treated mice, even though some potential difference in the capacity to maintain retinoid homeostasis may exist between the two strains, based on our qPCR analysis. However, in this study, β-carotene was only administered acutely (single dose). Thus, the effects of prolonged provitamin A availability on the retinoid status of this organ remain to be established.

Remarkably, the absence of *Ldlr* resulted in the ~50% reduction in hepatic β-carotene accumulation; this was unlikely due to enhanced metabolism of the provitamin A carotenoid in this organ, as assessed by qPCR analysis of the two β-carotene cleavage enzymes *Bco1* and *Bco2.* Upon β-carotene availability, the expression levels of *Bco1* were similar in the liver of *Ldlr*^+/−^ and *Ldlr*^−/−^ dams, suggesting that the ability to metabolize intact β-carotene by symmetric cleavage was unaffected by the complete lack of *Ldlr*. Nevertheless, hepatic *Bco1* was upregulated in response to β-carotene availability but only in the *Ldlr*^+/−^. Although it seems reasonable that the liver may enhance its ability to convert the available β-carotene into retinoids for storage, retinoid levels were similar in the liver of *Ldlr*^+/−^ vehicle- and β-carotene-injected dams [37,38]. Moreover, the β-carotene-mediated upregulation of hepatic *Bco1* was not observed in wild-type dams β-carotene-supplemented with a similar protocol [17]. Thus, we cannot exclude a genotype-specific effect on *Bco1* expression that could also possibly explain the increased baseline expression levels of the β-carotene symmetric cleavage gene *Bco1* in the *Ldlr*^−/−^ livers. Further studies are needed to confirm this hypothesis and clarify the underlying molecular mechanisms. The hepatic transcription of *Bco2*, induced by carotenoids in vivo [8,19,39], was also not affected by β-carotene availability in the liver of the *Ldlr*^−/−^ dams. Our data suggest a potential upregulation of the asymmetric cleavage pathway in the liver of the *Ldlr*^+/−^ dams. However, this effect unlikely contributed to the difference in hepatic β-carotene content between *Ldlr*^+/−^ and *Ldlr*^−/−^ dams. Together, these data point, for the first time, to a critical role of the LDL receptor in facilitating the uptake of β-carotene in the livers of adult pregnant mice. In agreement with this hypothesis, *Ldlr* mRNA levels increased about 30% in the liver of wild-type dams supplemented with a single dose of β-carotene (data not shown). It is also noteworthy that the hepatic uptake of circulating β-carotene is not fully abrogated in the absence of LDLr, clearly suggesting that, for example, other lipoprotein receptors such as LRP1 and VLDLr, that can take up LDL, albeit less efficiently than LDLr, may assist in cellular internalization of β-carotene by the hepatocytes. Indeed, changes in liver-specific expression of these lipoprotein receptors during gestation have previously been reported in mice lacking LDLr [40]. Interestingly, in a study by Crawford et al. [41], a diet containing β-carotene in combination with other antioxidants such as vitamin E and ascorbic acid contributed towards a reduction in the fatty streak lesions in the aortic sinus in the hearts of *Ldlr*^−/−^ mice fed an atherogenic diet. Based on our data, we could speculate that in *Ldlr*^−/−^ mice, β-carotene not taken up by the liver could accumulate in the heart, among other tissues, and may contribute towards the reduction of atherogenic phenotype such as fatty streak lesions in the heart. Further studies investigating the underlying mechanism of action of β-carotene in the heart would be of great interest.

Contrary to the liver, the lack of difference in β-carotene accumulation in the placentas and embryos of the supplemented dams, as well as the similar retinoid levels, irrespective of the maternal-fetal *Ldlr* genotype, disfavor a possible involvement of either maternal or placental/embryonic LDLr in regulating embryonic β-carotene acquisition and its consequent local metabolism by the cleavage enzymes. In agreement with these observations, the embryos from the dams lacking *Ldlr* developed normally without congenital defects or mortality associated with vitamin A deficiency or excess, under this experimental condition. Currently, we cannot predict the effects of prolonged provitamin A availability on the retinoid status of placenta and embryo.

## 5. Conclusions

We provided evidence that LDLr contributes significantly to mediate β-carotene uptake in the adult mouse liver, at least during pregnancy, whereas it does not seem to play a major role in placental and embryonic uptake of the provitamin A. In humans, several mutations (over 1000) in the *Ldlr* gene have been identified. Although severity of these mutations in causing hypercholesterolemia varies greatly, many of these lead to a several-fold elevation in the serum cholesterol [42,43,44]. Moreover, in humans, apoE—a structural component of lipoproteins that modulate cholesterol metabolism by binding to various lipoprotein receptors—exists in three isoforms (E2, E3 and E4) that bind to LDLr with specific affinity (E4 > E3 > E2) [45]. Interestingly, recent data suggest that dietary β-carotene metabolism is modulated by the *apoE* genotype, especially in the liver [46]. Thus, we speculate that gene variations in the *Ldlr* and/or *apoE* may play a crucial role in the hepatic uptake of carotenoids incorporated into lipoproteins. In the case of vitamin A deficiency, such populations may be at a greater need of effective β-carotene supplementation strategies, which may be different from the ones utilized in the case of populations with normal lipid metabolism.

## Figures and Tables

**Table 1:** Maternal plasma lipoprotein lipid and β-carotene concentrations in *Ldlr*^+/−^ and *Ldlr*^−/−^ pregnant dams supplemented with β-carotene.

Lipoprotein Fraction	TC (mg/dL)	TG (mg/dL)	BC (μg/dL)
		*Ldlr^+/−^+ BC*	
VLDL + Chylo	10 ± 2	10 ± 3	9 ± 6
LDL + IDL	59 ± 13	10 ± 4	121 ± 95
HDL	92 ± 8	9 ± 1	384 ± 105
Whole serum	159 ± 14	27 ± 7	538 ± 190
		*Ldlr*^−/−^*+ BC*	
VLDL + Chylo	34 ± 11 *	16 ± 3 *	30 ± 16 *
LDL + IDL	91 ± 16 *	13 ± 2	265 ± 169
HDL	68 ± 50	12 ± 2	15; 402
Whole serum	208 ± 8 *	30 ± 4	446 ± 107

Data are mean ± SD, except when *n* = 2 (individual values are indicated). *n* = 3–4 dams/group; statistical analysis by a Students’ *t*-test or Mann–Whitney test as per data distribution; * *p* < 0.05 vs. corresponding fraction in *Ldlr*^+/−^ + BC. BC, β-carotene; TC, total cholesterol; TG, triglycerides.

**Table 2:** β-carotene, retinol and retinyl ester concentrations in the livers of *Ldlr*^+/−^ and *Ldlr*^−/−^ pregnant mice with or without β-carotene administration

Maternal Genotype and Treatment	*n*	BC (μg/g)	ROH (μg/g)	RE (μg/g)
*Ldlr*^+/−^ + Veh	4	n.d.	7 ± 1	448 ± 171
*Ldlr*^+/−^ + BC	7	104 ± 46	6 ± 1	856 ± 354
*Ldlr*^−/−^ + Veh	4	n.d.	6 ± 3	686 ± 183
*Ldlr*^−/−^ + BC	7	42 ± 23 *	6 ± 1	649 ± 139

Data are mean ± SD; statistical analysis by ANOVA; *, *p* < 0.05 vs. *Ldlr*^+/−^ within the same treatment group. n.d., not detected (below detection limits). BC, β-carotene; ROH, retinol; RE, retinyl esters.

**Table 3:** β-carotene in the placentas and embryos of *Ldlr^+/−^* and *Ldlr^−/−^* pregnant mice administered β-carotene.

Maternal Genotype and Treatment	Placental/Embryonic Genotype	*n*	Placental BC (ng/g)	Embryonic BC (ng/g)
*Ldlr^+/−^* + Veh	*Ldlr^+/−^*	8	n.d.	n.d.
	*Ldlr^−/−^*	6	n.d.	n.d.
*Ldlr^+/−^* + BC	*Ldlr^+/−^*	13	581 ± 147	13 ± 5
	*Ldlr^−/−^*	13	590 ± 158	18 ± 8
*Ldlr^−/−^* + Veh	*Ldlr^+/−^*	10	n.d.	n.d.
	*Ldlr^−/−^*	10	n.d.	n.d.
*Ldlr^−/−^* + BC	*Ldlr^+/−^*	6	558 ± 187	19 ± 10
	*Ldlr^−/−^*	6	529 ± 99	19 ± 10

Data are mean ± SD; *n* = 1–5 placentas or embryos/dam (3–4 dams/genotype); statistical analysis by ANOVA. BC, **β**-carotene; Veh, vehicle.
